# Exploring the Incentive Function of Virtual Academic Degrees in a Chinese Online Smoking Cessation Community: Qualitative Content Analysis

**DOI:** 10.2196/42260

**Published:** 2023-07-04

**Authors:** Yuxing Qian, Zhenghao Liu, Edmund W J Lee, Yixi Wang, Zhenni Ni

**Affiliations:** 1 School of Information Management Wuhan University Wuhan China; 2 Center for Studies of Information Resources Wuhan University Wuhan China; 3 Big Data Institute Wuhan University Wuhan China; 4 Wee Kim Wee School of Communication and Information Nanyang Technological University Singapore Singapore; 5 School of Journalism and Communication Renmin University of China Beijing China

**Keywords:** online smoking cessation community, motivational affordances, virtual academic degrees, digital incentives, content analysis

## Abstract

**Background:**

Previous studies on online smoking cessation communities (OSCCs) have shown how such networks contribute to members’ health outcomes from behavior influence and social support perspectives. However, these studies rarely considered the incentive function of OSCCs. One of the ways OSCCs motivate smoking cessation behaviors is through digital incentives.

**Objective:**

This study aims to explore the incentive function of a novel digital incentive in a Chinese OSCC—the awarding of academic degrees—to promote smoking cessation. It specifically focuses on “Smoking Cessation Bar,” an OSCC in the popular web-based Chinese forum Baidu Tieba.

**Methods:**

We collected discussions about the virtual academic degrees (N= 1193) from 540 members of the “Smoking Cessation Bar.” The time frame of the data set was from November 15, 2012, to November 3, 2021. Drawing upon motivational affordances theory, 2 coders qualitatively coded the data.

**Results:**

We identified five key topics of discussion, including members’ (1) intention to get virtual academic degrees (n=38, 2.47%), (2) action to apply for the degrees (n=312, 20.27%), (3) feedback on the accomplishment of goals (n=203, 13.19%), (4) interpersonal interaction (n=794, 51.59%), and (5) expression of personal feelings (n=192, 12.48%). Most notably, the results identified underlying social and psychological motivations behind using the forum to discuss obtaining academic degrees for smoking cessation. Specifically, members were found to engage in sharing behavior (n=423, 27.49%) over other forms of interaction such as providing recommendations or encouragement. Moreover, expressions of personal feelings about achieving degrees were generally positive. It was possible that members hid their negative feelings (such as doubt, carelessness, and dislike) in the discussion.

**Conclusions:**

The virtual academic degrees in the OSCC created opportunities for self-presentation for participants. They also improved their self-efficacy to persist in smoking cessation by providing progressive challenges. They served as social bonds connecting different community members, triggering interpersonal interactions, and inducing positive feelings. They also helped realize members’ desire to influence or to be influenced by others. Similar nonfinancial rewards could be adopted in various smoking cessation projects to enhance participation and sustainability.

## Introduction

### Background

Smoking cessation is one of the core topics in public health globally. Practitioners and scholars in this field are concerned about how to better design and implement smoking cessation intervention projects [1-3]. With the increasing public awareness of the harm of smoking and the popularization of social networking services, online smoking cessation communities (OSCCs) are developing rapidly. OSCCs are places that overcome spatiotemporal constraints, provide participants with diverse resources, and create a positive environment to encourage smoking cessation [4]. Many smokers participate in OSCCs to ask questions, share their experiences, set goals, and record their cessation progress [5-7]. Additionally, some OSCCs are ground-up initiatives formed by smokers who want to quit smoking and are not led by smoking intervention projects developed by public health and health promotion practitioners.

There are different theoretical perspectives undertaken by public health and health promotion scholars in examining why OSCCs are important for encouraging and helping members quit smoking. From the behavior influence perspective, scholars stress the positive influence of a smoke-free web-based environment in OSCCs. More specifically, they have investigated OSCC members’ web-based social relationships [8,9] and how peer-to-peer internet networks and subjective norms positively influenced members’ self-efficacy, attitudes, and behavior about smoking cessation [4,6,10-13]. For example, a prior study employed social capital theory and found that structural capital (social ties) and relational capital (reciprocity) motivated members’ knowledge-sharing behavior in OSCCs [6]. Another study found that smokers’ exposure to peers’ positive sentiment about nicotine replacement therapy (NRT) in an OSCC was positively related to their offline NRT use [4].

From the social support perspective, scholars have highlighted the richness and benefits of social support from OSCCs. Numerous studies analyzed the content of posts in OSCCs to explore the types (such as emotional, informational, and esteem), distribution (such as under different cessation stages), and perceptions of social support available in OSCCs [5,14,15]. For example, guided by social support theory and the behavior stages theoretical transformation model, one study conducted a multidimensional content analysis on posts and replies in a Chinese OSCC. It found that various types of social support were available in the OSCC, and members could receive different compositions of social support in different scenarios and behavior stages to meet their needs accurately [5].

In summary, previous studies showed that OSCCs could provide a smoke-free web-based environment that positively influences members’ perceptions, attitudes, and behaviors toward smoking cessation and enable them to exchange various social support to better overcome difficulties in quitting smoking. However, those studies rarely considered the incentive function of OSCCs. Incentives are key elements that can enhance participants’ motivation (both intrinsic and extrinsic) and impact the perdurability and extensibility of smoking cessation projects [16,17]. Financial incentives, known as a typical kind of extrinsic motivation, are offered most often in traditional projects [18,19]. For example, project managers give participants rewards (cash, voucher, or gift) or help participants calculate the money they saved by quitting smoking [20,21]. However, the effect of these financial incentives is controversial because they may only be effective for the duration of time that the incentives are offered [18].

As for OSCCs, we observed that participants could get nonmonetary digital rewards instead of financial incentives. For example, in the “Smoking Cessation Bar,” an OSCC on the web-based Chinese forum Baidu Tieba dedicated to discussing smoking cessation, members are granted academic degrees depending on their duration of smoking cessation. These virtual academic degrees imitate the academic degrees in the real world, including bachelor’s, master’s, and doctoral degrees from low to high, with a postdoctoral certificate regarded as the highest degree level in this OSCC. They were initially put forward and designed by the OSCC manager. The longer an OSCC member keeps a record of quitting, the higher degree they can get. The activity of virtual degrees has been carried out since March 7, 2007. According to the statistical results of degree applications in the “Smoking Cessation Bar,” there were 1836 members awarded bachelor’s degrees, 1255 members awarded master’s degrees, 772 members awarded doctor’s degrees, and 152 members awarded the postdoctoral certificate as of January 17, 2022.

However, currently, we know very little about these virtual academic degrees, and their incentive function is still unclear. What do members think of these academic degrees? Could such digital rewards motivate members of the OSCC to maintain smoking cessation? Given the popularity and persistence of this activity, it is noteworthy for public health and health promotion practitioners and scholars because the incentive function of these virtual academic degrees has the potential to expand into other OSCCs, even offline smoking intervention projects. Members’ discussions on the academic degrees allow for assessing topics related to virtual academic degrees. Based on the discussion content, scholars can explore the incentive function of virtual academic degrees with the qualitative research method [22], which has been widely used in web-based content analyses [23-25].

Accordingly, this study aims to explore and understand the incentive function of virtual academic degrees. It adds to the existing literature by exploring the mechanisms of OSCCs from a relatively new perspective that can strengthen members’ adherence to the OSCC and improve their health outcomes.

### Theoretical Framework: Motivational Affordances

**Figure 1:**
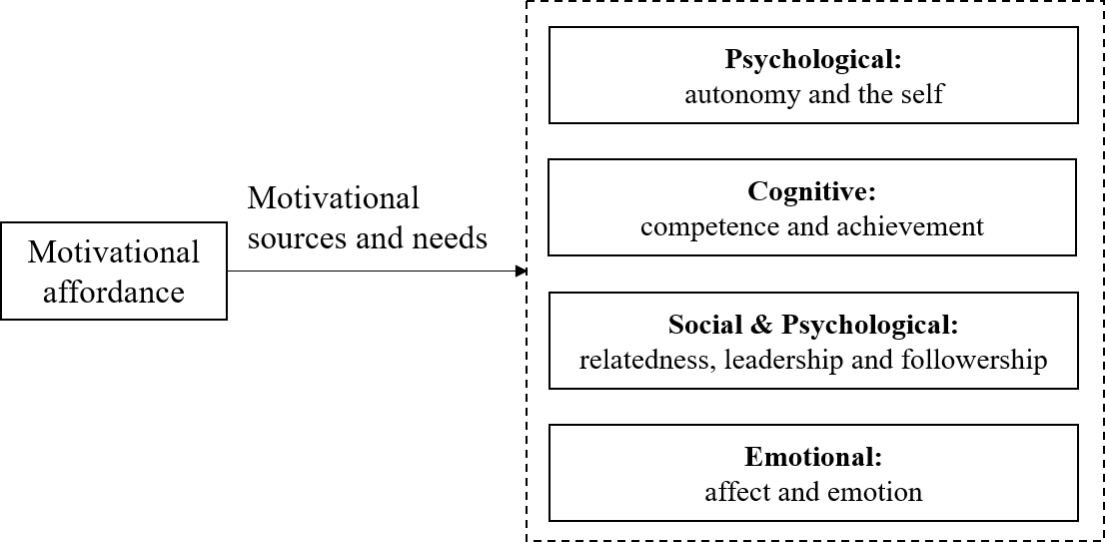
The motivational affordances theory.

We employed the motivational affordances theory to explore how virtual academic degrees present an incentive function. This theory extends the self-determination theory (which focuses on intrinsic motivation including competence, autonomy, and relatedness) [26]. According to the motivational affordances theory [27], information and communication technologies (ICTs) have motivational affordances that can be divided into several areas of motivational sources and needs, as shown in Figure 1. Therefore, the theory can serve as a framework to guide the design of ICTs to attract potential consumers, increase consumer loyalty, and stimulate continued use [27]. It has been applied in various contexts [28-30] and is especially suitable for the context of OSCCs because online communities are typical of ICTs [31,32].

As defined in the motivational affordances theory, motivational sources and needs cover four areas that include (1) *autonomy and the self*; (2) *competence and achievement*; (3) *relatedness*, *leadership, and followership*; and (4) *affect and emotion*.

*Autonomy* means members’ psychological need to make choices at the beginning and their behavior regulation, while the *self* indicates members’ psychological well-being about defining and creating the self, specifically self-identity. The need for *competence* appears when we carry out a task with a certain difficulty and complexity that precisely fits our current skills. *Achievement* refers to someone who accomplished a goal and is satisfied with it. *Relatedness* reflects people’s psychological need to belong and can be cultivated via human interaction. *Leadership and followership* involve and satisfy people’s need for influencing others and being influenced by others. *Affect and emotion* refers to inducing users’ emotions via both the biological system (by initial exposure to ICTs) and the cognitive system (by intensive cognitive activities) [27].

Guided by the motivational affordances theory, our study could help better elucidate the mechanism of OSCCs in aiding in smoking cessation and support the future development of web-based smoking intervention projects. In addition, exploring the relationship between reward and user engagement could yield actionable insights relevant to the gamification of behavior support solutions in web-based platforms. We are mainly concerned with the following research questions: (1) What are the key topics of members’ discussion on obtaining academic degrees for smoking cessation? (2) What do these topics of discussion tell us about the motivational affordance of virtual academic degrees as an incentive for smoking cessation?

## Methods

### Ethical Considerations

Because we used secondary web-based open-access data, ethical approval was not required by our institutional ethics boards.

### Data Source and Processing

Overall, this qualitative study followed the JARS-Qual (Journal Article Reporting Standards for Qualitative Research) suggested by Levitt et al [33].

To answer our research questions, we collected data from the “Smoking Cessation Bar” on Baidu Tieba dedicated to discussing smoking cessation [34]. Baidu Tieba is a widely used web-based Chinese forum. It can be accessed via PC or mobile phone via the internet and uses forums as a place for members to interact socially on a specific topic. As of January 17, 2022, the “Smoking Cessation Bar” had amassed 717,902 members, with 274,447 posts and 10,844,398 replies. It can be considered influential among smokers and is an excellent data source of research on member interaction.

**Figure 2:**
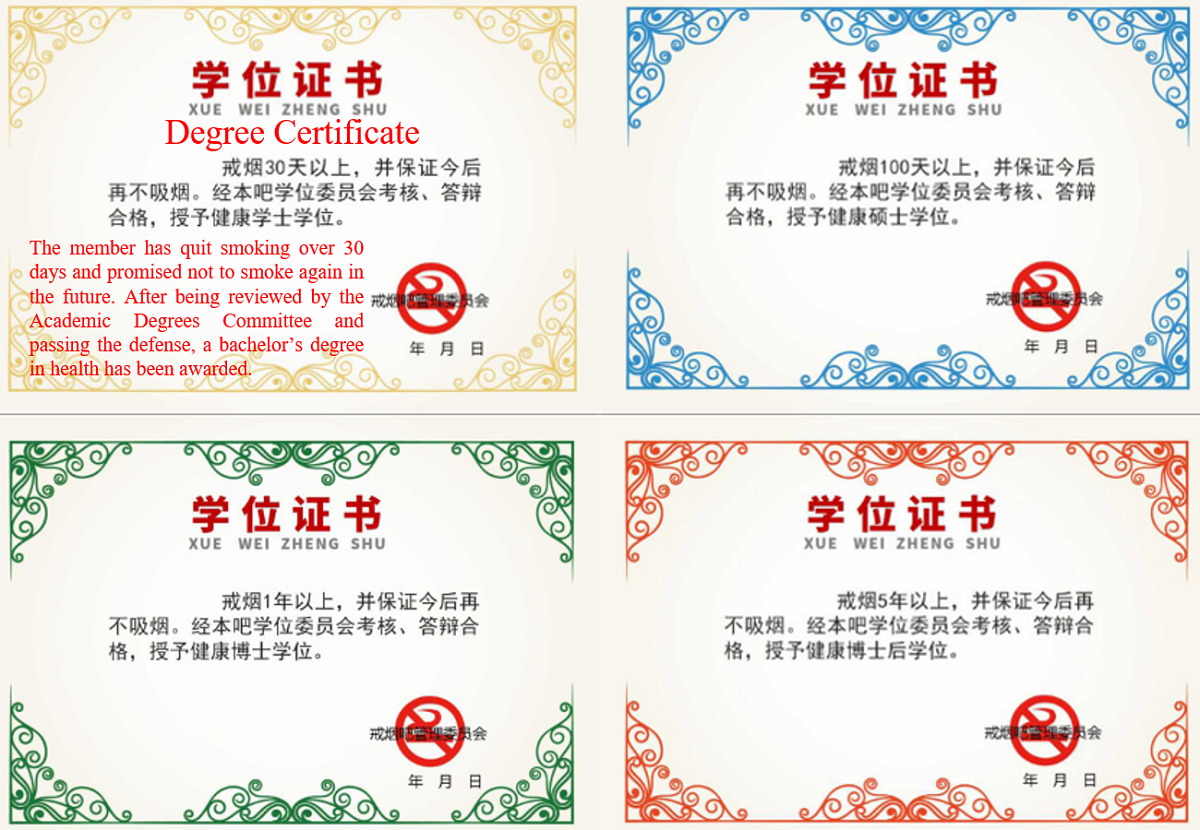
Examples of the virtual degree.

**Figure 3:**
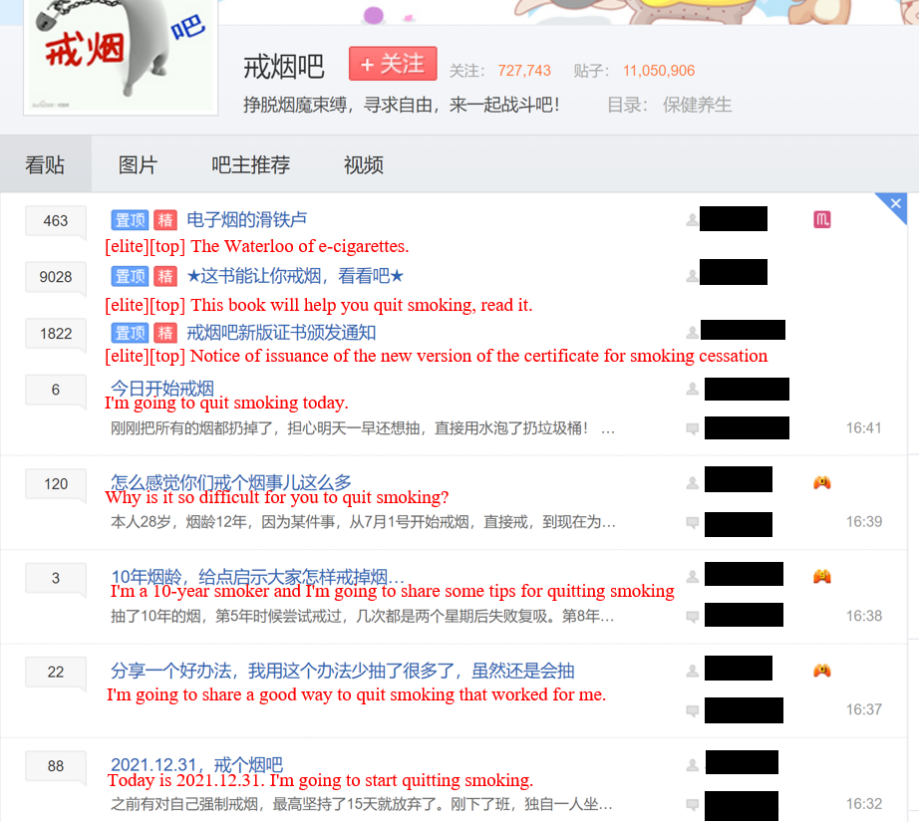
Home page of the “Smoking Cessation Bar."

**Figure 4:**
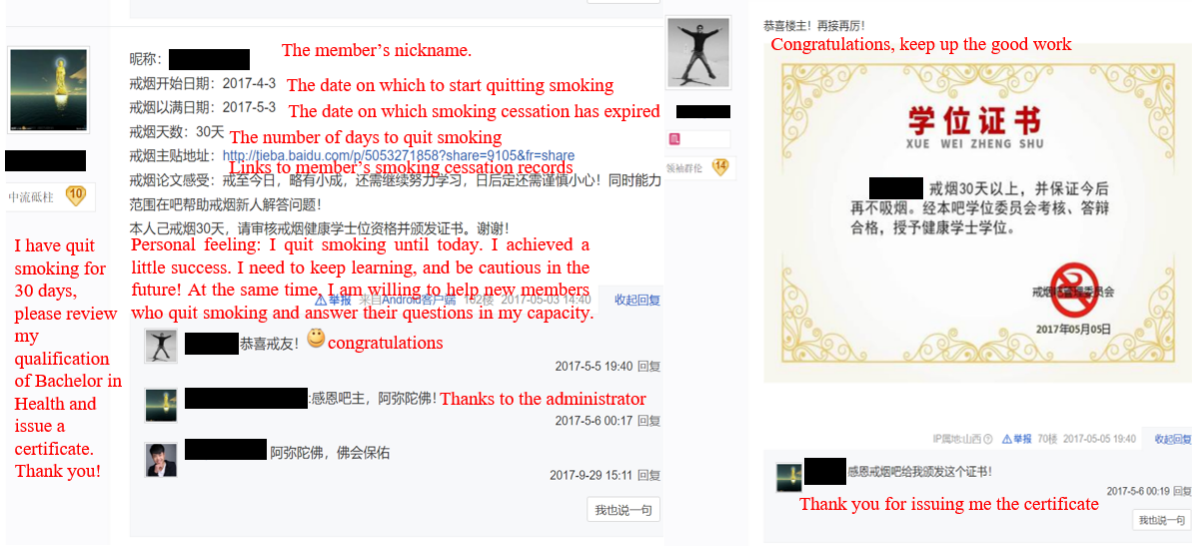
An example of applying for and awarding a bachelor’s degree certificate.

Members who want to apply for the virtual degree are asked to create a post for self-report and update this post via daily check-in. The member’s smoking cessation duration is presented in the post. When the duration reaches 30 days, members are requested to submit the link to their self-report posts to the management committee. After the committee approves, members are awarded a bachelor’s degree. They can further apply for the master’s degree, doctor’s degree, and postdoctoral certificate when the duration reaches 100 days, 1 year, and 5 years, respectively. Examples of the virtual degree are shown in Figure 2, with the statement in the bachelor’s degree translated into English. Figures 3 and 4 are screenshots of the home page and posts of the “Smoking Cessation Bar,” respectively. We have blinded certain information for privacy purposes, even though this is a public page.

To accurately collect members’ feedback on the academic degrees, we determined some related keywords, including *degree*, *certificate*, *bachelor*, *master*, and *doctor*. Then, we searched for these keywords separately in the forum and crawled the returned results with a script written in Python (version 3.7; Python Software Foundation). If these keywords existed in a record’s title, post content, or reply content, the record would show in returned results. Next, we merged each search result into a data set, deleted duplicate records, and removed 416 uncorrelated records by manual checking. Finally, we retained 1193 records (published by 540 different members) as the corpus for further content analysis. The time frame of the data set was from November 15, 2012, to November 3, 2021.

### Content Analysis and Statistics

To apply the motivational affordances theory in our context, we determined the 4 aforementioned areas of motivational sources and needs as the base framework. Then, according to the guidance of content analysis in the online community context by Pfeil and Zaphiris [35], 2 coders (authors ZL and YW) with experience conducting content analysis read records one by one in the corpus to gain familiarity with the raw data and gain a complete understanding of the text content. We determined a single phrase as the unit of analysis because a single phrase is enough to convey a specific concept. After that, the 2 coders independently put forward the first set of codes according to their impressions of keywords or sentences related to the 4 areas. The coders were asked to give the definition and examples of each code. Next, we summarized and compared these codes. We merged the same or similar codes and omitted or adjusted codes that were vague, ambiguous, or that had subordinate relationships. After refining these codes, we allocated each code into the corresponding area and formed the primary and secondary categories with clear definitions. Finally, the 2 coders reached an agreement through discussion, and the coding schema was formed, as shown in Table 1.

**Table 1:** Coding schema and definition.

Area, primary category, and secondary category				Definition
**Psychological: autonomy and the self**				
	**1. Intention**			
		1.1. Counseling method to apply for a degree	Members want to know how to get a degree.	
	**2. Action**			
		2.1. Applying for a degree	Members apply for the degree.	
		2.2. Asking about the reason for not receiving a degree	Members did not receive the degree and asked the reason for it.	
**Cognitive: competence and achievement**				
	**3. Goal accomplishment**			
		3.1. Pride	Members express their pride in receiving a degree.	
		3.2. Shame	Members have received the degree but fail to maintain smoking cessation.	
		3.3. Regret	Members express their regret about not maintaining smoking cessation for their degree.	
		3.4. Self-encouragement	Members motivate themselves to maintain their smoking cessation for their degree.	
**Social and psychological: relatedness, leadership, and followership**				
	**4. Interaction**			
		4.1. Congratulation	Members congratulate others on their degrees.	
		4.2. Praise	Members praise others that have a high degree.	
		4.3. Encouragement	Members encourage others to keep on and apply for a higher degree.	
		4.4. Notification	Members inform others of the way to get a degree.	
		4.5. Sharing	Members share their perceptions or stories.	
		4.6. Recommendation	Members suggest others read the self-report of the higher degree holder.	
**Emotional: affect and emotion**				
	**5. Personal feelings**			
		5.1. Anticipation	Members look forward to receiving a degree.	
		5.2. Joy	Members feel joyful participating in the activity of pursuing the degree.	

To measure the intercoder reliability, we randomly selected 50 pieces of records from the corpus, and the 2 coders (authors ZL and YW) labeled them independently. The reliability coefficient was calculated by the formula proposed by Perreault [36]. When there are many ways that codes are observed, this formula can help to correct the reliability coefficient. In the following equation, *I_r_* represents the reliability, *F*_0_ represents that judgment of coders is consistent, *N* represents the total amount of codes judged by coders, and *k* indicates the different ways that codes are observed:



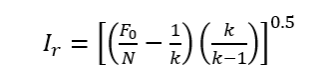



The reliability coefficient was 0.91, reflecting that the reliability of the coding schema was reasonable. After coding all records, we performed descriptive statistics analysis with R software (version 3.6.1; R Foundation for Statistical Computing) to count the frequency and percentage of each code.

## Results

### Overview

Using qualitative content analysis of members’ discussion, we identified five key topics, including members’ (1) intention to get virtual academic degrees, (2) action to apply for the degrees, (3) feedback on goal accomplishment, (4) interpersonal interaction, and (5) expression of personal feelings. These topics embody areas of motivational sources and needs in the context of virtual academic degrees in the OSCC. Each code’s number and percentage are shown in Table 2.

**Table 2:** Coding results.

Primary and secondary categories		Amount, n (%)
**1. Intention**		38 (2.47)
	1.1. Counseling method to apply for a degree	38 (2.47)
**2. Action**		312 (20.27)
	2.1. Applying for a degree	295 (19.17)
	2.2. Asking the reason for not receiving a degree	17 (1.11)
**3. Goal accomplishment**		203 (13.19)
	3.1 Pride	43 (2.79)
	3.2 Shame	12 (0.78)
	3.3 Regret	16 (1.04)
	3.4 Self-encouragement	132 (8.58)
**4. Interaction**		794 (51.59)
	4.1 Congratulations	117 (7.60)
	4.2 Praise	79 (5.13)
	4.3 Encouragement	81 (5.26)
	4.4 Notification	48 (3.12)
	4.5 Sharing	423 (27.49)
	4.6 Recommendation	46 (3.99)
**5. Personal Feelings**		192 (12.48)
	5.1 Anticipation	87 (5.66)
	5.2 Joy	105 (6.82)

There were 1539 codes in total. We found that 874 (73.26%) records had 1 code, 294 (24.64%) records had 2 codes, 23 (1.93%) records had 3 codes, and 2 (0.17%) records had 4 codes. At the primary category level, codes of *interaction* accounted for the highest proportion (n=794, 51.59%), followed by *action* (n=312, 20.27%) and *goal accomplishment* (n=203, 13.19%).

*Intention* and *action* reflect *autonomy and the self*. Codes of *intention* indicated members’ willingness to participate in getting virtual academic degrees. They asked about the method to get the degrees, such as:

How can I apply for this degree? Who issues it?

Members’ intentions were not often proactively expressed by posting. Codes of *intention* had the lowest number (n=38, 2.47%). Instead, members’ actions, interactions, and feelings were often reflected in posts, as indicated by the higher number of posts. Codes of *action* were of 2 types. The first one was the statement of applying for a degree (n=295, 19.17%), such as:

I have quit smoking for more than 30 days, please reward me a bachelor’s degree and issue a certificate.

Second, members who did not successfully earn their degree inquired about the reasons (n=17, 1.11%), like the following:

It has been 104 days since I quit smoking, and I have not received the master’s degree certificate. I do not know why.

*Goal accomplishment* reflected *competence and achievement*. Codes of *goal accomplishment* included members’ feedback on how well they achieved their goals. When it went well, they mainly expressed pride (n=43, 2.79%) and self-encouragement (n=132, 8.58%). In some cases, members also showed negative feedback (including shame and regret) because they failed to obtain a certificate or relapsed after obtaining a certificate. For example:

I smoked again tonight and felt dizzy and nauseous. I regretted it deeply.

I got a bachelor’s degree, unfortunately, relapse again, it is simply too humiliating.

*Relatedness, leadership, and followership* can be formed by *interaction*. We found that the virtual degrees facilitated various types of interaction among members. Sharing was the most common way of interaction. Members frequently shared their feelings and experiences with others (n=423, 27.49%), such as:

After a hundred days of quitting smoking, we became masters. The remnants of the Smoke Demon who survived the first battle between man and Smoke were also upgraded to the backbone.

Interaction between members also conveys emotional support, including congratulations (n=117, 7.60%), praise (n=79, 5.13%), and encouragement (n=81, 5.26%). Some post examples are as follows:

Congratulations on your degree certificate!

…Learn from you! I want to get a postdoctoral certificate too.

There are many people who got doctor’s degrees and postdoctoral certificates, waiting for you to receive them! Come on!

Other types of interactions accounted for less.

*Personal feelings* convey *affect and emotion*. Codes of *personal feelings* reflected 2 types of members’ positive emotional feedback toward the virtual academic degrees. Generally, members recognized the sense of accomplishment and happiness that the virtual academic degrees brought them by expressing positive feelings like anticipation (n=87, 5.65%) and joy (n=105, 6.82%). For example:

Already 31 days, I began to prepare to apply for a degree certificate, and I was a little excited to think about it.

I got my master’s degree, very happy.

I’m also a guy with a degree now, ha-ha.

Check in every day, don’t make any excuses for relapse, come on!

## Discussion

### Principal Findings

In this study, we used the motivational affordances theory to understand the incentive function of virtual academic degrees in an OSCC by identifying motivational sources and needs in member discussions. Based on our results, virtual academic degrees present incentive functions in different areas of motivational sources and needs.

Briefly, the virtual academic degrees in the OSCC create opportunities for self-presentation for participants. They also improve their self-efficacy to persist in smoking cessation by providing progressive challenges. They serve as social bonds connecting different community members, trigger interpersonal interactions, and induce positive feelings. Virtual academic degrees realize members’ desire to influence or to be influenced by others. In these ways, they can arouse members’ intrinsic motivation [30].

First, virtual academic degrees highlight members’ autonomy and reflect their self-identity. According to the forum rules, members’ intentions and actions for getting degrees are their voluntary responses to the activity instead of following the mandatory instruction of the OSCC manager. Additionally, the degrees are displayed in the members’ self-report posts that others can see. Thus, they support members’ needs for defining and representing the self and allow members to decide how they want to express themselves. 

Second, the hierarchy of virtual academic degrees sets different challenge levels for members because different levels of the degrees correspond to different cessation periods. Members may have different skills, knowledge, and experience to overcome difficulties during smoking cessation. Thus, the hierarchy of virtual academic degrees can cover all possible targeted users and helps members set milestones. This challenge increases members’ self-efficacy and confidence to achieve long-term smoking cessation goals [37]. As indicated by our results, getting the virtual academic degrees brought members a sense of achievement. In the progress of goals achievement, many members expressed self-encouragement toward their goals. Regret and shame were expressed when members relapsed. Negative feedback was also meaningful because it reflected members’ desire to achieve certain goals.

Third, virtual academic degrees showed prominent incentive functions in the *social and psychological* area, including *relatedness, leadership, and followership*. As an official activity organized by the OSCC, participation in the virtual academic degree activity means that the member is involved in a group with shared interests and goals. The member has more chances to interact with others and strengthen their relationships. Our results showed that various kinds of member interactions could convey emotional (like praise, congratulation, and encouragement) and informational support (like sharing, notification, and recommendation), facilitating closer relationships among members. These kinds of interactions conveyed supportive, motivational messages that contributed to members’ satisfaction with their user experience [38]. Furthermore, the degree level only depends on the member’s smoking cessation duration. Higher degree holders are considered to have determination, persistence, and experience quitting smoking. Thus, they can get compliments from other members (eg, “*Postdoc, ha, great*!” and “*another Ph.D., congratulations, learn from you*”). They also actively encourage and support other members (eg, “*You can get a bache*lor’s *degree. I am already a doctor”* and “*the Doctor is a role model for those who come after him”*). Most importantly, members can impact others by sharing their experience and knowledge or receiving positive mentorship from higher degree holders (such as receiving recommendations). These interactions can promote information exchange, trigger members’ observational learning [39], and facilitate their continuous participation in the OSCC for a further goal. Over time, the members are continuously exposed to a positive smoke-free environment that positively influences their attitudes and behavior [4,10,11]. In summary, virtual academic degrees facilitate members’ interaction, represent members’ social bonds, and enhance their relatedness.

Finally, for *affect and emotion,* members expressed their feelings of anticipation when beginning their pursuit of the virtual degrees and joy when they met the condition of a certificate. In contrast to other web-based learning environments [40], we did not identify negative personal feelings (such as doubt, carelessness, dislike, anxiety, boredom, and frustration) in members’ discussions. One possible explanation could be that expressing such negative personal feelings could cause conflicts among members. Members with negative feelings might not have wanted to damage their social relationships within the community and therefore did not express negative feelings. Moreover, there might be a junction between *emotion and affect* and *competence and achievement*. On the one hand, some codes (such as *pride* and *shame*) were allocated to *goal accomplishment*. According to the theory, they are in line with the meaning of *competence and achievement*, although they are also presented by emotional discussion content. On the other hand, positive personal feelings usually accompanied successful goal accomplishment (eg, “*My smoking quitting is very successful. I enjoy it for 66 days. I am so happy*”).

### Implications

One theoretical implication of our study is that it tried to understand OSCCs’ mechanism from the incentive function perspective. In contrast, prior literature on OSCCs was usually posited from behavior influence and social support perspectives to demonstrate how OSCCs contribute to members’ health outcomes. To the best of our knowledge, this is the first study to investigate the incentive function of virtual academic degrees in the context of OSCCs, suggesting the value and benefits of OSCCs in promoting members to quit smoking.

Second, this study expanded the application of the motivational affordances theory. In previous studies, this theory was usually used to determine variables in quantitative studies [41,42] or discussions surrounding the ICT design framework [40,43,44]. As for qualitative research, this theory was applied in wearable information systems [45] but was not widely used in such research methods. In this study, we examined key topics of discussion through the lens of motivation to explore how virtual degrees present an incentive function. Our study showed the applicability and effectiveness of this theory in the OSCC context, thus paving the way for its subsequent use in other similar online community contexts. Public health and health promotion scholars are also suggested to apply the motivational affordances theory in other health promotion settings (such as dietary intervention and chronic disease monitoring).

As for practical implications, our study started with the members’ discussion and used a bottom-up analysis to demonstrate the incentive function of virtual academic degrees that represent a kind of nonfinancial digital reward. It suggests the value and benefits of OSCCs in promoting members to quit smoking and has the potential to inspire the design and practice of various smoking cessation projects to enhance their participation and sustainability with such nonfinancial rewards.

For other OSCCs, the operators can set up similar nonfinancial digital rewards to the virtual academic degrees in the “Smoking Cessation Bar.” Members with higher academic degrees have achieved long-term smoking cessation, indicating that they have more knowledge and practical experience in smoking cessation. The connotation of virtual academic degrees is consistent with real-world academic degrees (indicating someone is an expert with more knowledge and experience in a specific domain). Considering that the existing real-world academic degree system is used globally, the virtual academic degree also has the potential to be generalized to other cultural contexts. Scholars and project managers can also design other kinds of digital rewards more acceptable and valued by the locals, as we only included a Chinese OSCC in this study. Other digital rewards might also make sense and are worthy of consideration. Project managers should also notice how these digital rewards drive member engagement. In the “Smoking Cessation Bar,” the level of the degree earned by members only depends on the duration of smoking cessation (ie, the days members reported in their posts). Other kinds of online engagement (such as publishing a new post or making a comment) do not contribute to getting the degree. This rule reinforces the importance of smoking cessation duration and helps guarantee the quality of the content in the OSCC because members are averted from posting meaningless or irrelevant content when applying for a degree. In addition to the virtual academic degrees for long-term members, project managers can also provide some certificates for those members who only keep quitting for a short term (like 7 days or 14 days), such as “junior student” and “senior student.” This will provide more accessible digital rewards and may help attract more members to be involved in the activity. In this way, the incentive function of these digital rewards can make sense in an earlier stage. Members will build social relationships and be influenced by others during short-term smoking cessation. Thus, it is also possible for them to evolve from short-term to long-term users.

Next, virtual academic degrees can also tap into the gamification design of some smoking cessation apps. Compared with the existing achievement system (such as badges and levels) in these apps [37,46,47], virtual academic degrees extend previous gamification strategies by taking the form of academic degrees that exist in the real world. In this way, a sense of achievement from the real world is transferred to an OSCC, making earning a virtual degree more serious than a recreational activity. This may increase members’ perceived value of virtual academic degrees. Indeed, the primary function of many smoking cessation apps (such as Kwit and Quit Genius) is self-management, and our study found that members’ interaction greatly contributes to the incentive function of virtual academic degrees. App designers can add functions for users to better present their digital rewards and promote interpersonal interactions. 

Moreover, for offline smoking cessation projects, program planners and managers can add measures similar to virtual academic degrees to their projects. For example, they can award participants digital or physical certificates when conducting group interventions [48-50] or NRT [51,52]. In this way, participants’ efforts and persistence in quitting smoking can be correlated with the certificates, conveying a sense of honor and achievement. As a supplement to financial incentives (such as cash and vouchers), these low-cost strategies can make such programs more welcoming and improve their efficiency by arousing participants’ intrinsic motivations.

Finally, we also need to pay attention to supervising digital rewards. Some members may cheat and lie about the period they quit smoking, which can lead to misdirected incentives and negatively impact health-related outcomes [53]. We cannot fully collect online users’ data to prove their real smoking cessation period. Although it is difficult to completely avoid cheating if the user insists on doing so, community managers can cultivate a community culture of mutual assistance and honest self-report to reduce the occurrence of cheating. For example, in the OSCC, users with high-level virtual academic degrees (doctor’s degrees or above) can be encouraged to establish communication subgroups to mentor lower degree holders and new users. Additionally, managers can that suggest low-level or new users join communication groups to receive more support and guidance. Evaluation and verification from friends can also help prevent cheating and promote authenticity. For example, if a member has truly ceased smoking for a certain period, their friends may be aware of this personal situation. They can respond to the member's post, providing evaluation and verification to support their application for the virtual degree. Through these measures, high-level users can play an exemplary role that helps increase their influence and involvement in the community, and members can form subgroups to strengthen their interactions and build closer, more genuine relationships.

### Limitations and Future Directions

There are some limitations to this study. First, we could not trace all related discussions for content analysis because of the retrieval restriction in the Baidu Tieba platform (which only returns up to 76 pages of retrieved results). Some posts may have also been deleted by members and managers or removed due to technical issues. In addition, members engaged in a very wide range of discussions within the OSCC. These reasons made the proportion of discussion related to virtual academic degrees low. Second, although our study includes discussions about virtual academic degrees in a Chinese OSCC, it is restricted by data source; demographic characteristics such as gender, age, educational background, and household characteristics were unavailable. These factors may affect members’ attitudes toward virtual degrees. Future research can cover these factors using surveys, interviews, and behavioral experiments and compare groups to offer more evidence and insights into how these degrees motivate OSCC members. Future research can also pay attention to members’ negative feelings about the degrees, which were not observed in this study, to further understand members’ attitudes toward the degree. Finally, considering the potential junction between *affect and emotion* and *competence and achievement*, future research can explore the inner relationship between these 2 areas.

## Abbreviations

ICT: information and communication technology
JARS-Qual: Journal Article Reporting Standards for Qualitative Research
NRT: nicotine replacement therapy
OSCC: online smoking cessation community
